# Supplementary material for: Heat adaptation of phage T7 under an extended genetic code
Source: Virus Evol. 2021 Dec 1;7(2):veab100. doi: 10.1093/ve/veab100 (PMC8923235; doi:10.1093/ve/veab100)
Supplement: veab100_Supp [file veab100_supp.zip › Sequencing_Analysis.zip!/Sequencing_Analysis/S1/evidence/RA_30.html]

BRESEQ :: Evidence


| Read alignment evidence... | | | | | | | | | | | |
| --- | --- | --- | --- | --- | --- | --- | --- | --- | --- | --- | --- |
|  | seq id | position | | ref | new | freq | score (cons/poly) | reads | annotation | genes | product |
| \* | NC\_001604.1 | 34,968 | 0 | C | A | 64.9% | 2014.2 / inf | 3835 | intergenic (–/–) | *–/–* | –/– |
| Reads supporting (aligned to +/- strand):  **ref** base C (689/653);  **new** base A (1253/1227);  **total** (1943/1892) | | | | | | | | | | | |
| Fisher's exact test for biased strand distribution *p*-value = 6.35e-01 | | | | | | | | | | | |
| Kolmogorov-Smirnov test that lower quality scores support variant *p*-value = 4.79e-01 | | | | | | | | | | | |

Only 100 of 3853 total aligned reads displayed.  

`CTCCAATCGTGATTTTAATATCCCGTTTGAGTATCTAGCCCGTAAGTTCGTAGTGGTAACTCTTATTGGTGTAGACCGAAAGGTCCTTACGATTAATACAGACTATCGCTTTGCTACACGTACTACTATCTCTCTGACAAAGGCTTGGGGTCCAGCCGATGGCTACACGACCATCGAGTTACGTCGAGTAACCTCCACTACCGACCGATTGGTTGACTTTACGGATGGTTCAATCCTCCGCGCGTATGACCTTAACGTCGCTCAGATTCAAACGATGCACGTAGCGGAAGAGGCCCGTGACCTCACTACGGATACTATCGGTGTCAATAACGATGGTCACTTGGATGCTCGTGGTCGTCGAATTGTGAACCTAGCGAACGCCGTGGATGACCGCGATGCTGTTCCGTTTGGTCAACTAAAGACCATGAACCAGAACTCATGGCAAGCACGTAATGAAGCCTTACAGTTCCGTAATGAGGCTGAGACTTTCAGAAACCAAGCGGAGGGCTTTAAGAACGAGTCCAGTACCAACGCTACGAACACAAAGCAGTGGCGCGATGAGACCAAGGGTTTCCGAGACGAAGCCAAGCGGTTCAAGAATA  >  NC_001604.1/34668‑35269`  

                                                                                                                                                                                                                                                                                                            `|`                                                                                                                                                                                                                                                                                                               
   `cAATCGTGATTTTAATATCCCGTTTGAGTATCTAGCCCGTAAGTTCGTAGTGGTAACTCTTATTGGTGTAGACCGAAAGGTCCTTACGATTAATACAGACTATCGCTTTGCTACACGTACTACTATCTCTCTGACAAAGGCTTGGGGTCCAGCCGATGGCTACACGACCATCGAGTTACGTCGAGTAACCTCCACTACCGACCGATTGGTTGACTTTACGGATGGTTCAATCCTCCGCGCGTATGACCTTAACGTCGCTCAGATTCAAACGATGCACGTAGCGGAAGAGGCCCGTGAACTc                                                                                                                                                                                                                                                                                                            <  1:388669/301‑1 (MQ=255)`  
      `tCGTGATTTTAATATCCCGTTTGAGTATCTAGCCCGTAAGTTCGTAGTGGTAACTCTTATTGGTGTAGACCGAAAGGTCCTTACGATTAATACAGACTATCGCTTTGCTACACGTACTACTATCTCTCTGACAAAGGCTTGGGGTCCAGCCGATGGCTACACGACCATCGAGTTACGTCGAGTAACCTCCACTACCGACCGATTGGTTAACTTTACGGATGGTTCAATCCTCCGCGCGTATGACCTTAACGTCGCTCAGATTCAAACGATGCACGTAGCGGAAGAGGCCCGTGAACTCACt                                                                                                                                                                                                                                                                                                         <  1:148027/301‑1 (MQ=255)`  
          `taTTTTAATATCCCGTTTGAGTATCTAGCCCGTAAGTTCGTAGTGTTAACTCTTATTGGTGTAGACCGAAAGGTCCTTACGATTAATACAGACTATCGCTTTGCTACACGTACTACTATCTCTCTGACAAAGGCTTGGGTTCCAGCCGATGGCTACACGACCATCGAGTTACGTCGAGTAACCTCCACTACCGACCGATTGGTTGACTTTACGGATGGTTCAATCCTCCGCGCGTATGACCTTAACGTCGCTCAGATTTAAACGATGCACGTAGCGGAAGAGGCCCGTGACCTCACTACgg                                                                                                                                                                                                                                                                                                     <  1:227346/300‑1 (MQ=255)`  
             `ttaaaTATCCCGTTTGAGTATCTAGCCCGTAAGTTCGTAGTGGTAACTCTTATTGGTGTAGACCGAAAGGTCCTTACGATTAATACAGACTATCGCTTTGCTACACGTACTACTATCTCTCTGACAAAGGCTTGGGGTCCAGCCGATGGCTACACGACCATCGAGTTACGTCGAGTAACCTCCACTACCGACCGATTGGTTGACTTTACGGATGGTTCAATCCTCCGCGCGTATGACCTTAACGTCGCTCAGATTCAAACGATGCACGTAGCGGAAGAGGCCCGTGAACTCACTACGGATa                                                                                                                                                                                                                                                                                                  <  1:166557/298‑1 (MQ=255)`  
                 `gagacaggTTTGAGTATCTAGCCCGTAAGTTCGTAGTGGTAACTCTTATTGGTGTAGACCGAAAGGTCCTTACGATTAATACAGACTATCGCTTTGCTACACGTACTACTATCTCTCTGACAAAGGCTTGGGGTCCATCCGATGGCTACACGACCATCGAGTTACGTCGAGTAACCTCCACTACCGACCGATTGGTTGACTTTACGGATGGTTCAATCCTCCGCGCGTATGACCTTAACGTCGCTCAGATTCAAACGATGCACGTAGCGGAAGAGGCCCGTGACCTCACTACGGATACTAt                                                                                                                                                                                                                                                                                              <  1:407312/294‑1 (MQ=255)`  
                 `ataaCCCGTTTGGGTATCTATCCCGTAAGTTCGTAGTGGGAACTCTTATTGGTGTAGACCGAAAGGTCCTTACGATTAATACAGACTATCGCTTTGCTACACGTACTACTAGCTCTCTGACAAAGGCTTGGGGTCCAGCCGATGGCTACACGACCATCGAGTTACGTCGAGTAACCTCCACTACCGACCGATTGGTTAACTTTACGGATGGTTCAATCCTCCGCGCGTATGACCTTAACGTCGCTCAGATTCAAACGATGCACGTAGCGGAAGAGGCCCGTGACCTCACTACGGATACTAt                                                                                                                                                                                                                                                                                              <  1:265598/301‑1 (MQ=255)`  
                      `ccGTTTGAGTATCTAGCCAGTAAGTTCGTAGTGGTAACTCTTATTGGTGTAGACCGAAAGGTCCTTACGATTAATACAGACTATCGCTTTGCTACACGTACTACTATCCCTCTGACAAAGGCTTGGGGTCCAGCCGATGGCTACACGACCATCGAGTTACGTCGAGTAACCTCCACTACCGACCGATTGGTTGACTTTACGGATGGTTCAATCCTCCGCGCGTATGACCTTAACGTCGCTCAGATTCAAACGATGCACGTAGCGGAAGAGGCCCGTGAACTCACTACGGATACTATCGgtg                                                                                                                                                                                                                                                                                         <  1:319607/301‑1 (MQ=255)`  
                             `aGTATCTAGCCCGTAAGTTCGTAGTGGTAACTCTTATTGGTGTAGACCGAAAGGTCCTTACGATTAATACAGACTATCGCTTTGCTACACGTACGACTATCTCTCTGACAAAGGATTGGGGTCCAGCCGATGGCTACACGACCATCGAGTTACGTCGAGTAACCTCCACTACCGACCGATTGGTTGACTTTACGGATGGTTCAATCCTCCGCGCGTATGACCTTAACGTCGCTCAGATTCAAACGATGCACGTAGCGGAAGAGGCCCGTGAACTCACTACGGATACTATCGGTGTCAATaa                                                                                                                                                                                                                                                                                  <  1:342196/301‑1 (MQ=255)`  
                               `aaTCTAGCCCGTAAGTTCGTAGTGGTAACTCTTATTGGTGTAGACCGAAAGGTCCTTACGATTAATACAGACTATCGCTTTGCTACACGTACTACTATCTCTCTGACAAAGGCTTGGGGTCCAGCCGATGGCTACACGACCATCGAGTTACGTCGAGTAACCTCCACTACCGACCGATTGGTTGACTTTACGGATGGTTCAATCCTCCGCGCGTATGACCTTAACGTCGCTCAGATTCAAACGATGCACGTAGCGGAAGAGGCCCGTGACCTCACTACGGATACTATCGGTGTCAATAACg                                                                                                                                                                                                                                                                                <  1:463344/300‑1 (MQ=255)`  
                                        `cGTAAGTTCGTAGTGGTAACTCTTATTGGTGTAGACCGAAAGGTCCTTACGATTAATACAGACTATCGCTTTGCTACACGTACTACTATCTCTCTGACAAAGGCTTGGGGTCCAGCCGATGGCTACACGACCATCGAGTTACGTCGAGTAACCTCCACTACCGACCGATTGGTTGACTTTACGGATGGTTCAATCCTCCGCGCGTATGACCTTAACGTCGCTCAGATTCAAACGATGCACGTAGCGGAAGAGGCCCGTGACCTCACTACGGATACTATCGGTGTCAATAACGATGGTCACt                                                                                                                                                                                                                                                                       <  1:232395/301‑1 (MQ=255)`  
                                            `gtgtataagagacagctctTATTGGTGTAGACCGAAAGGTCCTTACGATTAATACAGACTATCGCTTTGCTACACGTACTACTATCTCTCTGACAAAGGCTTGGGGTCCAGCCGATGGCTACACGACCATCGAGTTACGTCGAGTAACCTCCACTACCGACCGATTGGTTGACTTTACGGATGGTTCAATCCTCCGCGCGTATGACCTTAACGTCGCTCAGATTCAAACGATGCACGTAGCGGAAGAGGCCCGTGAACTCACTACGGATACTATCGGTGTCAATAACGATGGTCACTTGGa                                                                                                                                                                                                                                                                   <  1:646478/286‑1 (MQ=255)`  
                                                     `tGGTAACTCTTATTGGTGTAGACCGAAAGGTCCTTACGATTAATACAGACTATCGCTTTGCTACACGTACTACTATCTCTCTGACAAAGGCTTGGGGTCCAGCCGATGGCTACACGACCATCGAGTTACGTCGAGTAACCTCCACTACCGACCGATTGGTTAACTTTACGGATGGTTCAATCCTCCGCGCGTATGACCTTAACGTCGCTCAGATTCAAACGATGCACGTAGCGGAAGCTGCCCGTGACCTCACTACGGATCCTATCGGTGTCAATAACGACTGTCTCttctacacatctcc                                                                                                                                                                                                                                                          >  1:377209/1‑289 (MQ=255)`  
                                                           `ctctTATTTGTGTAGACCGAAAGGTCCTTACGATTAATACAGACTATTGCTTTGCTACACGTACTACTATCTCTCTGACAAAGGCTTGGGGTCCAGCCGATGGCTACACGACCATCGAGTTACGTCGAATAACCTCCACTACCGACCGATTGGTTGACTTTACGGATGGTTCAATCCTCCGCGCGTATGACCTTAACGTCGCTCAGATTCAAACGATGCACGTAGCGGAAGAGGCCCGTGAACTCACTACGGATACTATCGGTGTCAATAACGATGGTCACTTGGATGCTCGTGGtcgtcg                                                                                                                                                                                                                                                    >  1:172256/1‑301 (MQ=255)`  
                                                           `ctctTATTGGTGTAGACCGAAAGGTCCTTACGATTAATACAGACTATCGCTTTGCTACACGTACTTCTATCTCTCTGACAAAGGCTTGGGGTCCAGCCGATGGCTACACGACCATCGAGTTACGTCGAGTAACCTCCACTACCGACCGATTGGTTGACTTTACGGATGGTTCAATCCTCCGAGCGTATGACCTTAACGTCGCTCAGATTCAAACGATGCACGTAGCGGAAGAGGCCCGTGACCTCACTACGGATACTATCGGTGTCAATAACGATGGTCACTTGGATGCTCGTGGtcgtcg                                                                                                                                                                                                                                                    >  1:253997/1‑301 (MQ=255)`  
                                                               `tATTGGTGTAGACCGAAAGGTCCTTACGATTAATACAGACTATCGCTTTGCTACGCGTACTACTATCTCTCTGACAAAGGCTTGGGGTCCAGCCGATGGCTACACGACCATCGAGTTACGTCGAGTAACCTCCACTACCGACCGATTGGTTGACTTTACGGATGGTTCAATCCTCCGCGCGTATGACCTTAACGTCGCTCAGATTCAAACGATGCACGTAGCGGAAGAGGCCCGTGAACTCACTACGGATACTCTCGGTGTCAATAACGATGGTCACTTGGATGCTCGTGGTCGTCTAAtt                                                                                                                                                                                                                                                >  1:507214/1‑301 (MQ=255)`  
                                                               `tATTGGTGTAGACCGAAAGGTCCTTACGATTAATACAGACTATCGCTTTGCTACACGTACTACTATCTCTCTGACAAAGGCTTGGGGTCCAGCCGATGGCTACACGACCATCGAGTTACGTCGAGTAACCTCCACTACCGACCGATTGGTTGACTTTACGGATGGTTCAATCCTCCGCGCGTATGACCTTAACGTCGCTCAGATTCAAACGATGCACGTAGCGGAAGAGGCCCGTGAACTCACTACGGATACTATCGGTGTCAATAACGATGGTCACTTGGATGCTCGTGGTCGTCGAAtt                                                                                                                                                                                                                                                >  1:569328/1‑301 (MQ=255)`  
                                                                   `ggTGTAGACCGAAAGGTCCTTACGATTACTACACACTATCGCTTTGCTACACGTACTACTATCTCTCTGACAAAGGCTTGGTGTCCAGCCGATGGCTACACGACCATCAAGTTACTTCGAGTAATCTCCACTACCGACCGATTTGTTTACTTTACGGATGGTTCCATCCTCCGCGCGTACCACCTTAACGTCGCTCAGAGTTAAACGATGCACGTTGCGGAAGACTCCCGTGAACTCACTCCGGATTCTATCGGTGTCAATACTGCTTGTCCATTGGATGCTCGTGCTCGTCGtgttgttt                                                                                                                                                                                                                                            >  1:301799/1‑299 (MQ=255)`  
                                                                                          `gATTAATACAGACTATCGCTTTGCTACACGTACTACTATCTCTCTGACAAAGGCTTGGGGTCCAGCCGATGGCTACACGACCATCGAGTTACGTCGAGTAACCTCCACTACCGACCGATTGGTTGACTTTACGGATGGTTCAATCCTCCGCGCGTATGACCTTAACGTCGCTCAGATTCAAACGATGCACGTAGCGGAAGAGGCCCGTGACCTCACTACGGATACTATCAGTGTCAATAACGATGGTCACTTGGATGCTCGTGGTCGTCGAATTGTGAACCTAGCGAACGCCGTGGATGAc                                                                                                                                                                                                                     >  1:450793/1‑301 (MQ=255)`  
                                                                                            `taagagacagcTATCGCTTTGCTAAACGTACTACTATCTCTCTGACAAAGGCTTGGGGTCCAGCCGATGGCTACACGACCATCGAGTTACGTCGAGTAACCTCCACTACCGACCGATTGGTTGACTTTACGGATGGTTCAATCCTCCGCGCGTATGACCTTAACGTCGCTCAGATTCAAACGATGCACGTAGCAGAAGAGGCCCGTGAACTCACTACGGATACTATCGGTGTCAATAACGATGGTCACTTGGATGCTCGTGGTCGTCGAATTGTGAACCTAGCGAACGCCGTGGATGACcg                                                                                                                                                                                                                   <  1:602327/291‑1 (MQ=255)`  
                                                                                             `tAATACAGACTATCGCTTTGCTACACGTACTACTATCTCTCTGACAAAGGCTTGGGGTCCAGCCGATGGCTACACGACCATCGAGTTACGTCGAGTAACCTCCACTACCGACCGATTGGTTGACTTTACGGATGGTTCAATCCTCCGCGCGTATGACCTTAACGTCGCTCAGATTCAAACGATGCACGTAGCGGAAGAGGCCCGTTAACTCACTACGGATACTCTCGGTTTCAATAACGATTGTCCCTTGGATGCTCGTGGTCCTCGAATTGTGAACCTAGCGAACCCCGTGGATGAccac                                                                                                                                                                                                                  >  1:480495/1‑299 (MQ=255)`  
                                                                                               `aTACAGACTATCGCTTTGCTACACGTACTACTATCTCTCTGACAACGGCTTGGGGTCCAGCCGATGGCTACACGACCATCTAGTTACGTCTAGTAACCTCCACTACCGACCGATTTGTTGACTGTACGGATGGTTCAATCCTCCGCGCGTATGACCTTAACGTCGCTCAGATTCAAACGATGCCCGTAGCGTAAGTGGCCAGTGACCGCACTTCGGATACTCTCGGTGTCAATAACGATGGTCACTTGGATTCTCGTAGACGTCGCTTTGTGAACCTAGCGAACGCCGTGGATGACcgcgg                                                                                                                                                                                                                >  1:581340/1‑300 (MQ=255)`  
                                                                                               `aTACAGACTATCGCTTTGCTACACGTACTACTATCTCTCTGACAAAGGCTTGGGGTCCAGCCGATGGCTACACGACCATCGAGTTACGTCGAGTAACCTCCACTACCGACCGATTGGTTGACTTTACGGATGGTTCAATCCTCCGCGCGTATGACCTTAACGTCGCTCAGATTCAAACGATGCACGTAGCGGAAGAGGCCCGTGAACTCACTACGGATACTATCGGTGTCAATAACGATGGTCACTTGGATGCTCGTGGTCGTCGGATTGTGAACCTAGCGAACGCCGTGGATGACCGCGa                                                                                                                                                                                                                >  1:86153/1‑301 (MQ=255)`  
                                                                                               `aTACAGACTATCGCTTTGCTACACGTACTACTATCTCTCTGACAAAGGCTTGGGGTCCAGCCGATGGCTACACGACCATCGAGTTACGTCGAGTAACCTCCACTACCGACCGATTGGTTGACTTTAAGGATGGTTCAATCCTCCGCGCGTATGACCTTAACGTCGCTCAGATTCAAACGATGCACGTAGCGGAAGAGGCCCGTGAACTCCCTACGGATACTATCGGTGTCAATAACGCTGGTCACTTGGATGCGCGTGGTCGTCGAATTGTGAACCTAGCGAACGCCGTGGATGACCGCGa                                                                                                                                                                                                                >  1:52787/1‑301 (MQ=255)`  
                                                                                                    `gACTATCGCTTTGCTACACGTACTACTATCTCTCTGACAAAGGCTTGGGGTCCAGCCGATGGCTACACGACCATCGAGTTACGTCGAGTAACCTCCACTACCGACCGATTGGTTGACTTTACGGATGGTTCAATCCTCCGCGCGTATGACCTTAACGTCGCTCAGATTCAAACGATGCACGTAGCGGAAGAGGCCCGTGAACTCACTACGGATACTATCGGTGTCAATAACGATGGTCACTTGGATGCTCTTGGTCGTCGAATTGTGAACCTAGCGAACGCCGTGGATGACCGCGatgatg                                                                                                                                                                                                           >  1:657414/1‑301 (MQ=255)`  
                                                                                                    `gACTATCGCTTTGCTACACGTACTACTATCTCTCTGACAAAGGCTTGGGGTCCAGCCGATGGCTACACGACCATCGAGTTACGTCGAGTAACCTCCACTACCGACCGATTGGTTGACTTTACGGATGGTTCAATCCTCCGCGCGTATGACCTTAACGTCGCTCAGATTCAAACGATGCACGTAGCGGAAGAGGCCCGTGAACTCACTACGGATACTATCGGTGTCAATAACGATGGTCACTTGGATGCTCGTGGTCGTCGAATTGTGAACCTAGCGAACGCCGTGGATGAcctgtctctta                                                                                                                                                                                                           >  1:434750/1‑292 (MQ=255)`  
                                                                                                      `cTATCGCTTTGCTACACGTACTACTATCTCTCTGACAAAGGCTTGGGGTCCAGCCGATGGCTACACGACCATCGAGTTACGTCGAGTAACCTCCACTACCGACCGATTGGTTGACTTTACGGATGGTTCAATCCTCCGCGCGTATGACCTTAACGTCGCTCAGATTCAAACGATGCACGTAGCGGAAGAGGCCCGTGAACTCACTACGGATACTATCGGTGTCAATAACGATGGTCACTTGGATGCTCGTGGTCGTCGAATTGTGAACCTAGCGAACGCCGTGGATGACCGCGATGCTGtt                                                                                                                                                                                                         >  1:153766/1‑301 (MQ=255)`  
                                                                                                        `aTCGCTTTGCTACACGTACTACTATCTCTCTGACAAAGGCTTGGGGTCCAGCCGATGGCTACACGACCATCGAGTTACGTCGAGTAACCTCCACTACCGACCGATTGGTTGACTTTACGGATGGTTCAATCCTCCGCGCGTATGACCTTAACGTCGCTCAGATTCAAACGATGCACGTAGCGGAAGAGGCCCGTGAACTCACTACGGATACTATCGGTGTCAATAACGATGGTCACTTGGATGCTTGTGGTCGTCGAATTGTGAACCTAACGAAAGACGTGGATGACCGCGGTGCTGTTcc                                                                                                                                                                                                       >  1:667775/1‑301 (MQ=255)`  
                                                                                                           `gCTTTGCTACACGTACTACTATCTCTCTGACAAAGGCTTGGGGTCCAGCCGATGGCTACACGACCATCGAGTTACGTCGAGTAACATCCACTACCGACCGATTGGTTGACTTTAAGGATGGTTCAATCCTCCGCGCGTATGACCTTAACGTCGATCAGATTCAAACGATGCACGTAGCGGAAGAGGCCCGTGAACTCACTACGGATACTATCGGTGTCAATAACGATGGTCACTTGGATGCTCGTGGTCGTCGAATTGTGAACCTAGCGAACGCCGTGGATGACCGCGATGCTGTTCCGtt                                                                                                                                                                                                    <  1:469133/301‑1 (MQ=255)`  
                                                                                                            `cTTTGCTACACGTACTACTATCTCTCTGACAAAGGCTTGGGGTCCAGCCGATGGCTACACGACCATCGAGTTACGTCGAGTAACCTCCACTACCGACCGATTGGTTGACTTTACGGATGGTTCAATCCTCCGCGCGTATGACCTTAACGTCGCTCAGATTCAAACGATGCACGTAGCGGAAGAGGCCCGTGACCTCACTACGGATACTATCGGTGTCAATAACGATGGTCCCTTGGCTGCTCGTGGTCGTCGAATTGTGAACCTAGCGAACGCCGTGGATGCCCGCGATGCTGTTCCGttt                                                                                                                                                                                                   >  1:353386/1‑301 (MQ=255)`  
                                                                                                             `gagatgtgtagaggagacagctctctGACAAAGGCTTGGGGTCCAGCCGATGGCTACACGACCATCGAGGTACGTCGAGTAACCTCCACTACCGACCGATTGGTTGACGTTACGGATGGTTCAATCCTCCGCGCGTATGACCTTAACGTCGCTCAGATTCAAACGATGCACGTAGCGGAAGAGGCCCGTGAACTCACTACGGATACTATCGGTGTCAATAACGATGGTCACTTGGATGCTCGTGGTCGTCGAATTGTGAACCTAGCGAACGCCGTGGATGACCGCGATGCTGTTCCATTTg                                                                                                                                                                                                  <  1:129385/281‑1 (MQ=255)`  
                                                                                                                     `gacagctactaTCTCTCTGACAAAGGCTTGGGGTCCAGCCGATGGCTACACGACCATCGAGTTACGTCGAGTAACCTCCACTACCGACCGATTGGTTGACTTTACGGATGGTTCAATCCTCCGCGCGTATGACCTTAACGTCGCTCAGATTCAAACGATGCACGTAGCGGAAGAGGCCCGTGAACTCACTACGGATACTATCGGTGTCAATAACGATGGTCACTTGGATGCTCGTGGTCGTCGAATTGTGAACCTAGCGAACGCCGTGGATGACCGCGATGCTGTTCCGTTTGGTCAACTa                                                                                                                                                                                          <  1:27851/296‑1 (MQ=255)`  
                                                                                                                        `tactacTATCTCTCTGACAAAGGCTTGGGGTCCAGCCGATGGCTACACGACCATCGAGTTACGTCGAGTAACCTCCACTACCGACCGATTGGTTGACTTTACGGATGGTTCAATCCTCCGCGCGTATGACCTTAACGTCGCTCAGATTCAAACGATGCACGTAGCGGAAGAGGCCCGTGACCTCACTACGGATACTATCGGTGTCAATAACGATGGTCACTTGGATGCTCGTGGTCGTCGAATTGTGAACCTAGCGAACGCCGTGGATGACCGCGATGCTGTTCCGTTTGGTCAACTAAAg                                                                                                                                                                                       >  1:401407/1‑301 (MQ=255)`  
                                                                                                                        `tactacTATCTCTCTGACAAAGGCTTGGGGTCCAGCCGATGGCTACACGACCATCGAGTTACGTCGAGTAACCTCCACTACCGACCGATTGGTTGACTTTACGGATGGTTCAATCCTCCGCGCGTATGACCTTAACGTCGCTCAGATTCAAACGATGCACGTAGCGGAAGAGGCCCGTGACCTCACTACGGATACTATCGGTGTCAATAACGATGGTCACTTGGATGCTCGTGGTCGTCGAATTGTGAACCTAGCGAACGCCGTGGATGACCGCCATGCTGTTCCGTTTGGTCAACTAAAg                                                                                                                                                                                       >  1:550563/1‑301 (MQ=255)`  
                                                                                                                        `tactacTATCTCTCTGACAAAGGCTTGGGGTCCAGCCGATGGCTACACGACCATCGAGTTACGTCGAGTAACCTCCACTACCGACCGATTGGTTGACTTTACGGATGGTTCAATCCTCCGCGCGTATGACCTTAACGTCGCTCAGATTCAAACGATGCACGTAGCGGAAGAGGCCCGTGAACTCACTACGGATACTATCGGTGTCAATAACGATGGTCACTTGGATGCTCGTGGTCGTCGAATTGTGAACCTAGCGAACGCCGAGGATGCCCGCGATGCTGTTCCGTTTGGTCAACTAAAg                                                                                                                                                                                       >  1:326009/1‑301 (MQ=255)`  
                                                                                                                          `ctactaTCTCTTTGACAAAGGCTTGGGGTCCAGTCGATGGCTACACGACCATCGAGTTACGTCGAGTAACCTCCACTACCGACCGATTGGTTGACTTTACGGATGGTTCAATCCTCCGCGCGTATGACCTTAACGTCGCTCAGATTCAAACGAGGCACGTAGCGGAAGAGGCCCGTGAACTCACTACGGATACTATCGGTGTCAATAACGATGGTCACTTGGATGCTCGTGGTCGTCGAATTGTGAACCTAGCGAACGCCGTGGATGACCGCGATGCTGTTCCGTTTGGTCAACTAAAGAc                                                                                                                                                                                     <  1:91636/301‑1 (MQ=255)`  
                                                                                                                          `ctactaTCTCTCTGACAAAGGCTTGGGGTCCAGCCGATGGCTACACGACCATCGAGTTACGTCGAGTAACCTCCACTACCGACCGATTGGTTGACTTTACGGATGGTTCAATCCTCCGCGCTTATGACCTTAACGTCGCTCAGATTCAAACGATGCACGTAGCGGAAGAGGCCCGTGAACTCACTACGGATACTCTCGGTGACAATAACGAAGGTCACTTTGAACCTCGTGGTAGTCGAATTATGAATCTAGTCAACGCCGTGGATGACCGCTGTGCTGTTCCGTTTGGGCAACTAAAGAc                                                                                                                                                                                     >  1:456408/1‑301 (MQ=255)`  
                                                                                                                          `ctactaTCTCTCTGACAAAGGCTTGGGGTCCAGCCGATGGCTACACGACCATCGAGTTACGTCGAGTAACCTCCACTACCGACCGATTGGTTGACTTTACGGATGGTTCAATCCTCCGCGCGTATGACCTTAACGTCGCTCAGATTCAAACGATGCACGTAGCGGAAGAGGCCCGTGAACTCACTACGGATACTATCGGTGTCAATAACGATGGTCACTTGGATGCTCGTGGTCGTCGAATTGTGAACCTAGCGAACGCCGTGGATGACCGCGATGCTGTTCCGTTTGGTCAACTAAAGAc                                                                                                                                                                                     <  1:542610/301‑1 (MQ=255)`  
                                                                                                                          `ctaagaTCGCTCTGACAAAGGCTTGGGGTCCAGCCGATGGCTACACGACCATCGAGTTACGTCGAGTAACCTCCACTACCGACCGATTGGTTGACTTTACGGATGGTTCAATCCTCCGCGCGTATGACCTTAACGTCGCTCAGATTCAAACGATGCACGTAGCGGAAGAGGTCCGTGAACTCACTACGGATACTATCGGTGTCAATAACGATGGTCACTTGGATGCTCGTGGTCGTCGAATTGTGAACCTAGCGAACGCCGTGGATGACCGCGATGCTGTTCCGTTTGGTCAACTAAAGAc                                                                                                                                                                                     <  1:563442/296‑1 (MQ=255)`  
                                                                                                                          `atactaTCTCTCTGACAAAGGCGTGGGGTCCAGCCGATGGCTACACGACCATCGAGTTACGTCGAGTAACCTCCACTACCGACCGATTGGTTGACTTTACGGATGGTTCAATCCTCCGCGCGTATGACCTTAACGTCGCTCAGATTCAAACGATGCACGTAGCGGAAGAGGCCCGTGAACTCACTACGGATACTATCGGTGTCAATAACGATGGTCACTTGGATGCTCGTGGTCGTCGAATTGTGAACCTAGCGAACGCCGTGGATGACCGCGATGCTGTTCCGTTTGGTCAACTAAAGAc                                                                                                                                                                                     <  1:591222/300‑1 (MQ=255)`  
                                                                                                                               `aTCTCTAGGACAAAGGCTTGGGGTCCAGCCGGTGGCTACACGACCGTCGAGTTACGTCGAGTAACCACCACTACCGACCGATTGGTTGACTTTACGGATGGATCAATCCTCCGCGCGTATGACCTGAACGTCACTCAGATTCAAACGATGCACGTAGCGGAAGAGGCCCGTGAACTCACTACGGATACTATCGGTGTCAATAGCGATGGTCACTTGGATGCTCGTGGTCGTCGAATTGTGAACCTAGCGAACGCCGTGGATGACCGCGATGCTGTTCCGTTTGGTCAACTAAAGACCATgg                                                                                                                                                                                <  1:652657/301‑2 (MQ=255)`  
                                                                                                                                 `ctctctGACAAAGGCTTGGGATCCAGCCGATGGCTACACGACCATCGAGTTACGTCGAGTAACCTCCACTACCGACCGATTGGTTGACTTTACGGTTGGTTCAATCCTCCGCGCGTATGACCTTAACGTCGCTCAGATTCAAACGATGCACGTAGTGGAAGAGGCCCGTGAACTCACTACGGATACTATCGGTGTCAATAACGATGGTCACTTGGATGCTCGTGGTCGTCGAATTGTGAACCTAGCGAACGCCGTGGATGACAGCGATGCTGTTCCGTTTGGTCCACTAAATACCATGGAc                                                                                                                                                                              >  1:475929/1‑301 (MQ=255)`  
                                                                                                                                  `tccctGACAAAGGCTTGGGGTCCAGCCTATGGCTACACGACCATCGAGTTACGTCGAGTAACCTCCACTACCGACCGATTGGTTGACTTTACGGATGGTTCAATCCTCCGCGCGTATGACCTTAACGTCGCTCAGATTCAAACGATGCACGTAGCGGAAGAGGCCCGTGAACTCACTACGGATACTATCGGTGTCAATAACGATGGTCACTTGGATGCTCGTGGTCGTCGAATTGTGAACCTAGCGAACGCCGTGGATAACCGCGATGCTGTTCCGTTTGGTCAACTAAAGACCATGGAcc                                                                                                                                                                             <  1:313111/298‑1 (MQ=255)`  
                                                                                                                                   `ctctGACAAAGGCTTGGGGTCCAGCCGATGGCTACACGACCATCGAGTTACGTCGAGTAACCTCCACTACCGACCGCTTGGTTGACTTTACGGATGGTTCAATCCTCCGCGCGTATGACCTTAACGTCGCTCAGATTCAAACGATGCACGTAGCGGAAGAGGCCCGTGACCTCACTACGGATACTATCGGTTTCAATAACGATGGTCACTTGGATGCTCGTGGTCGTCGAATTGTGAACCTAGCGAACGCCGTTGATGCCCGCGATGCTGTTCCGTTTGGTCAACTAAATACCATGGACCa                                                                                                                                                                            >  1:621519/1‑301 (MQ=255)`  
                                                                                                                                       `gACAAAGGCTTGGGGTCCAGCCGATGGCTACACGACCATCGAGTTACGTCGAGTAACCTCCACTACCGACCGATTGGTTGACTTTACGGATGGTTCAATCCTCTGCGCGTATGACCTTAACGTCGCTCAGATTCAAACGATGCACGTAGCGGAAGAGGCCCGTGACCTCACTACGGATACTATCGGTGTCAATAACGATGGTCACTTGGATGCTCGTGGTCGTCGAATTGTGAACCTAGCGAACGCCGTGGATGACCGCGATGCTGTTCCGTTTGGTCAACTAAAGACCATGGACCAGCAc                                                                                                                                                                        >  1:189664/1‑301 (MQ=255)`  
                                                                                                                                       `gACAAAGGCTTGGGGTCCAGCCGATGGCTACACGACCATCGAGTTACGTCGAGTAACCTCCACTACCGACCGATTGGTTGACTTTACGGATGGTTCAATCCTCCGCGCGTATGACCTTAACGTCGCTCAGATTCAAACGATGCACGTAGCGGAAGAGGCCCGTGAACTCACTACGGATACTATCGGTGTCAATAACGATGGTCAATTGGATGCACGTGGTCTTCGAATTTTGAACCTAGCGAACGCCGTGGATGACCGCGATGCTGTTCCGTTTGGTCAACACAAGACCATTGACCAGAAc                                                                                                                                                                        >  1:57567/1‑301 (MQ=255)`  
                                                                                                                                       `gACAAAGGCTTGGGGTCCAGCCGATGGCTACACGACCATCGAGTTACGTCGAGTAACCTCCACTACCGACCGATTGGTTGACTTTACGGATGGTTCAATCCTCCGCGCGTATGACCTTAACGTCGCTAAGATTCAAACGATGCACGTAGCGGAAGAGGCTCGTGACCTCACTACGGATACTATCGGTGTCAATAACGATGGTCACTTGGATGCTCGTGGTCGTCGAATTGTGAACCTAGCGAACGCCGTGGATGACCGCGATGCTGTTCCGTTTGGTCAACTAAAGACCATGGACCAGaaa                                                                                                                                                                        >  1:439725/1‑300 (MQ=255)`  
                                                                                                                                            `aGGCTTTGGGTCCAGCCGATGGCTACACGACCATCGAGTTACGTCGAGTAACCTCCACTACCGACCGATTGGTTGACTTTACGGATGGTTCAATCCTCCGCGCGTATGACCTTAACGTCGCTCAGATTCAAACGATGCACGTAGCGGAAGAGGCCCGTGAACTCACTACGGATACTATCGGTGTCAATAACGATGGTCACTTGGATGCTCGTGGTCGTCGAATTGTGAACCTAGCGAACGCCGTGGATGACCGCGATGCTGTTCCGTTTGGTCAACTAAAGACCATGGACCAGAACTCATg                                                                                                                                                                   <  1:486995/301‑1 (MQ=255)`  
                                                                                                                                             `ggCTTGGGGTCCAGCCGATGGCTACACGACCATCGAGTTACGTCGAGTAACCTCCACTACCGACCGATTGGTTGACTTTACGGATGGTTCAATCCTCCGCGCGTATGACGTTAACGTCGCTCAGATTCAAACGATGCACGTAGCGGAAGAGGCCCGTGAACTCACTACGGATACTATCGGTGTCAATAACGATGGTCACTTGGATGCTCGTGGTCGTCGAATTGTGAACCTAGCGTACGCCGTGGATGACCGCGATGCTGTTCCGTTTGGTCAACTAAAGACCATGGACCAGAACTCATgg                                                                                                                                                                  <  1:215261/301‑1 (MQ=255)`  
                                                                                                                                               `cTTGGGGTCCAGCCGATGGCTACACGACCATCGAGTTACGTCGAGTAACCTCCACTACCGACCGATTGGTTGACTTTACGGATGGTTCAATCCTCCGCGCGTATGACCTTAACGTCGCTCAGATTCAAACGATGCACGTAGCGGAAGAGGCCCGTGAACTCACTACGGATACTATCGGTGTCAATAACGATGGTCACTTGGATGCTCGTGGTCGTCGAATTGTGAACCTAGCGAACGCCGTGGAAGACCGCGATGCTGTTCCGTTTGGTAAACTAAAGACCATGGACCAGAACTCATGGCa                                                                                                                                                                <  1:413895/301‑1 (MQ=255)`  
                                                                                                                                                          `gCCGATGGCTACACGACCATCGAGTTACGTCGAGTAACCTCCACTACCGACCGATTGGTTGACTTTACGGATGGTTCAATCCTCCGCGCGTATGACCTTAACGTCGCTCAGATTCAAACGATGCACGTAGCGGCAGAGGCCCGTGAACTCACTACGGATACTATCGGTGTCAATAACGATGGTCACTTGGATGCTCGTGGTCGTCGAATTGTGAACCTAGCGAACGCCGTGGATGACCGCGATGCTGTTCCGTTTGGTCAACTAAAGACCATGGACCAGAACTCATGGCAAGCACGTAAtt                                                                                                                                                     >  1:306903/1‑300 (MQ=255)`  
                                                                                                                                                          `gCCGATGGCTACACGACCATCGAGTTACGTCGAGTAACCTCCACTACCGACCGATTGGTTGACTTTACGGATGGTTCAATCCTCCGCGCGTATGACCTTAACGTCGCTCAGATTCAAACGATGAACGTAGCGGAAGAGGCCCGTGAACGCACTACGGATACTATCGGTGTCAATAACGATGGTCACTTGGATGCTCGTGGTCGTCGAATTGTGAACCTAGCGAACGCCGTGGATGACCGCGATGCTGTTCCGTTTGGTCAACTAAAGACCATGGACCAGAACTCATGGCAAGCACGTAATg                                                                                                                                                     <  1:49937/301‑1 (MQ=255)`  
                                                                                                                                                           `ccGATGGCTACACGACCATCGAGTTACGTCGAGTAACCTCCACTACCGACCGATTGGTTGACTTTACGGATGGTTCAATCCTCCGCGCGTATGACCTTAACGTCGCTCAGATTCAAACGATGCACGTAGCGGAAGAGGCCCGTGAACTCACTACGGATACTATCGGTGTCAATAACGATGGTCACTTGGATGCTCGTGGTCGTCGAATTGTGAACCTAGCGAACGCCGTGGATGACCGCGATGCTGTTCCGTTTGGTCAACTAAAGACCATGGACCAGAACTCATGGCAAGCACGTAATGa                                                                                                                                                    <  1:492206/301‑1 (MQ=255)`  
                                                                                                                                                            `cGATGGCTACACGAACATCGAGTTAGGTCGAGTAACCTCCACTACCGACCGATTGGTTGACTTTACGGATGGTTCAATCCTCCGAGCGTATGACCTTAACGTCGCTCAGATTCAAACGATGCACGTAGCGGAAGAGGCCCGTGAACTCACTACGGATACTATCGGTGTCAATAACGATGGTCACTTGGATGCTCGTGGTCGTCGAATTGTGAACCTAGCGAACGCCGTGGATGACCGCGATGCTGTTCCGTTTGGTCAACTAAAGACCATGGACCAGAACTCATGGCAAGCACGTAATGaa                                                                                                                                                   <  1:141400/301‑1 (MQ=255)`  
                                                                                                                                                             `aataagaaacggACCATCGAGTTACGTCGAGTAACCCCCACTACCGACCGATTGGTTGACTTTACGGATGGTTCAATCCTCCGCGCGTATGACCTTAACGTCGCTCAGATTCAAACGATGCACGTAGCGGAAGAGGCCCGTGAACTCACTACGGATACTATCGGTGTCAATAACGATGGTCACTTGGATGCTCGTGGTCGTCGAATGGTGAACCTAGCGAACGCCGTGGATGACCGCGATGCTGTTCCGTTTGGTCAACTAAAGACCATGGACCAGAACTCATGGCAAGCACGTAATGAAg                                                                                                                                                  <  1:427891/290‑1 (MQ=255)`  
                                                                                                                                                              `aaaggtaaccGACCATGGGGTTACGAC‑AATAACCTCCACTACCGACCGATTGGTTGACTTTACGGATGGTTCAATCCTCCGCGCGTATGACCTTAACGTCGCTCAGATTCAAACGATGCACGTAGCGGAAGAGACCCGTGAACTCACTACGGATACTATCGGTGTCAATAACGATGGTCACTTGGATGCTCGTGGTCGTCGAATTGTGAACCTAGCGAACGCCGTGGATGACCGCGATGCTGTTCCGTTTGGTCAACTAAAGACCATGGACCAGAACTCATGGCAAGCACGTAATGAAGcc                                                                                                                                                <  1:68273/292‑1 (MQ=255)`  
                                                                                                                                                               `tGGCTACACGACCATCGAGTTACGTCGAGTCACCTCCACTACCGACCGATTGGTTGACTTTACGGATGGTTCAATCCTCCGCGCGTATGACCTTAACGTCGCTCAGATTCAAACGATGCACGTAGCGGAAGAGGCCCGTGAACTCACTACGGATACTATCGGTGTCAATAACGATGGTCACTTGGATGCTCGTGGTCGTCGAATTGTGAACCTAGCGAACGCCGTGGATGACCGCGATGCTGTTCCGTTTGGTCAACTAAAGACCATGGACCAGAACTCATGGCAAGCACGTAATGAAGcc                                                                                                                                                <  1:220046/301‑1 (MQ=255)`  
                                                                                                                                                               `tGGCTACACGACCATCGAGTTACGTCGAGTAACCTCCACTACCGACCGATTGGTTAACTTTACGGATGGTTCAATCCTCCGCGCGTATGACCTTAACGTCGCTCAGATTCAAACGATGCACGTAGCGGAAGAGCCCCGTGACCTCACTACGGATACTATCGGTGTCAATAACGTTGGTCGCTTGGATGCTCGTGGTCGTCGAATTGTGAACCTAGCGAACGCCGTGGATGACCGCGATGCTGTTCCGTTTGGTTACCTGAAGACCATGGACCCGACATCATGGCCAGCACGACATGAAGcc                                                                                                                                                >  1:2480/1‑301 (MQ=255)`  
                                                                                                                                                                `ggCTACACGACCATCGAGTTACGTCGAGTAACCTCCACTACCGACCGATTGGTTGACTTTACGGATGGTTCAATCCTCCGCGCGTATGACCTTAACGTCGCTCAGATTCAAACGATGCACGTAGCGGAAGAGGCACGTGACCTCACTACGGAGACTATCGGTGTCAATAACGATGGTCACTTGGATGCTCGTGGTAGTCGAATTGTAAACCTAGCGAACGCCGTGGATGACCGCGATTCTGTTCCGTTTGGTCAACTAAAGACCATGGACCAGAACTCATGGCAAGCACGTCATGAGGcgt                                                                                                                                               >  1:364272/1‑299 (MQ=255)`  
                                                                                                                                                                  `cTACACGACCATCGAGTTACGTCGAGTAACCTCCACTACCGACCGATTGGTTGACTTTACGGATGGTTCAATCCTCCGCGCGTATGACCTTAACGTCGCTCAGATTCAAACGATGCACGTAGCGGAAGAGGCCCGTGACCTCACTACGGATACTATCGGTGTCAATATCGATGGTCACTTGGATGCTCGTGGTCGTCGAATTGTGAACCTAGCGAACGCCGTGGATGACCGCGATGCTGTTCCGTTTGGTCAACTAAAGACCATGGACCAGAACCCATGTCAAGAACGTAATGAAGCCttt                                                                                                                                             >  1:179667/1‑300 (MQ=255)`  
                                                                                                                                                                     `gagacagaTCGAGTTACGTCGAGTAACCTCCACTACCGACCGATTTGTTGACTTTACGGATGGTTCAATCCTCCGCGCGTATGACCTTAACGTCGCTCAGATTCAAACGATGCACGTAGCTGAAGAGGCCCGTGAACTCACTACGGATACTATCGGTGTCAATAACGATGGTCACTTGGATGCTCGTGGTCGTCGAATTGTGAACCTAGCGAACGCCGTGGATGACCGCGATGCTGTTCCGTTTGGTCAACTAAAGACCATGGACCAGAACTCATGGCAAGCACGTAATGAAGCCTTACAg                                                                                                                                          <  1:259989/294‑1 (MQ=255)`  
                                                                                                                                                                     `cacGACCATCGAGTTACGTCGAGTAACCTCCACTACCGACCGATTGGTTAACTTTACGGATGGTTCAATCCTCCGCGCGTATGACCTTAACGTCGCTCAGATTCAAACGATGCACGTAGCGGAAGAGGCCCGTGACCTCACTACGGATACTATCGGTGTCAATAACGATGGTCACTTGGATGCTCGTGGTCGTCGAATTGTGAACCTAGCGAACGCCGTGGATGACCGCGATGCTGTTCCGTTTGGTCAACTAAAGACCATGGACCAGAACTCATGGCAAGCACGTAATGAAGCCTTACAg                                                                                                                                          <  1:243854/301‑1 (MQ=255)`  
                                                                                                                                                                       `cGACCATCGAGTTACGTCGAGTAACCTCCACTACCGACCGCTTGGTTGACTTTACGGATGGTTCAATCCTCCGCGCGTATGACCTTAACGTCGCTCAGATTCAAACGATGCACGTAGCGGAAGAGGCCCGTGAACTCACTACGGCTACTATCGGTGTCAATAACGATGGTCACTTGGATGCTCGTGGTCGTCGAGTTGTTAACCTAGCGAACGCCGTGGATGACCGCGATGCTGTTCCGTTTGGTCTACTAAAGACCATGGACCAGAACTCTTGGCAAGCTCGTAATGAAGCCTTACAGtt                                                                                                                                        >  1:11690/1‑301 (MQ=255)`  
                                                                                                                                                                             `tCGAGTTACGTCGAGTAACCTCCACTACCGACCGATTGGTTGACTGTACGGATGGTTCAATCATCCGCGCGTATGACCTTAACGGCGCTCAGATTCAAACGATGCACGTAGCGGAAGAGGCACGTGAACTCACTACGGATACTATCGGTGTCAATAACGATGGTCACTTGGATGCTCGTGGTCGTCGAATTGTGAACCTAGCGAACGCCGTGGATGACCGCGATGCTGTTCCGTTTGGTCAACTAAAGACCATGGACCAGAACTCATGGCAAGCACGTAATGAAGCCTTACAGTTCCGTaa                                                                                                                                  <  1:111650/301‑1 (MQ=255)`  
                                                                                                                                                                             `tCGAGTTACGTCGAGTAACCTCCACTACCGACCGATTGGTTAACTTTACGGATGGTTCAATCCTCCGCGCGTATGACCTTAACGTCGCTCAGATTCAAACGATGCACGTAGCGGAAGAGGCCCGTGACCTCACTACGGATACTATCGGTGTCAATAACGATGGTCACTTGGATGCTCGTGGTCGTCGAATTGTGAACCTAGCGAACGCCGTGGATGACCGCGATGCTGTTCCGTTTGGTCAACTAAAGACCATGGACCAGAACTCATGGCAAGCACGTAATGAAGCCTTACAGTTCCGTaa                                                                                                                                  <  1:521122/301‑1 (MQ=255)`  
                                                                                                                                                                               `gtgtataagagacagaCCTCCACTACCGACCGATTGGTTGACTTTAAGGATGGTTCAATCCTCCGCGCGTATGACCTTAACGTCGCTCAGATTCAAACGATGCACGTAGCGGAAGAGGCCCGTGAACTCACTACGGATACTATCGGTGTCAATAACGATGGTCACTTGGATGCTCGTGGTCGTCGAATTGTGAACCTAGCGAACGCCGTGGATGACCGCGATGCTGTTCCGTTTGGTCAACTAAAGACCATGGACCAGAACTCATGGCAAGCACGTAATGAAGCCTTACAGTTCCGTAATg                                                                                                                                <  1:420225/286‑1 (MQ=255)`  
                                                                                                                                                                               `gtgTTACGTCGCGTAACCTCCACTACCGACCGAGTTGTTGACTTTACGGATGGTTCAATCCTCCGCGCGTATGACCTTAACGTCGCTCAGATTCAAACGATGCACGTAGCGGAAGAGGCCCGTGACCTCACTACGGATACTATCGGTGTCAATAACGATGGTCACTTGGATGCTCGTGGTCGTCGAATTGTGAACCTAGCGAACGCCGTGGATGACCGCGATGCTGTTCCGTTTGGTCAACTAAAGACCATGGACCAGAACTCATGGCAAGCACGTAATGAAGCCTTACAGTTCCGTAAtt                                                                                                                                <  1:55722/299‑2 (MQ=255)`  
                                                                                                                                                                               `gAGTTACGTCGAGTAACCTCCGCGACCGACCGATTGGTTGACTTTACGGATGGTGTAATACTCCGCGCGTATGACCTTAACGTCGCTCAGATTCAAACGATGCACGTAGCGGAAGAGGCCCGTGAACTCACTACGGATACTATCGGTGTCAATAACGATGGTCACTTGGATGCCCGTGGTCGTCGAATTGTGAACCTAGCGAACGCCGTGGATGACCGCGATGCTGTTCCGTTTGGTCAACTAAAGACCATGGACCAGAACTCATGGCAAGCACGTAATGAAGCCTTACAGTTCCGTAATg                                                                                                                                <  1:135632/301‑1 (MQ=255)`  
                                                                                                                                                                               `gAGTTACGTCGAGTAACCTCCACTACCGACCGATTGGTTGACTTTACGGATGGTTCAATCCTCCGCGCGTATGACCTTAACGTCGCTCAGATTCAAACGATGCACGTAGCGGAAGAGGCCCGTGACCTCACTACGGATACTATCGGTGTCAATAACGATGGTCACTTGGATGCTCGTGGTCGTCGAATTGTGAACCTAGCGAACGCCGTGGATGACCGCGATGCTGTTCCGTTTGGTCAACTAAAGACCATGGACCAGAACTCATGGCAAGCACGTAATGAAGCCTTACAGTTCCGTAATg                                                                                                                                <  1:348378/301‑1 (MQ=255)`  
                                                                                                                                                                                         `gAGTAACCTCCACTACCGACCGATTGGTTGACTTTACGGATGGTTCAATCCTCCGCGCGTATGACCTTAACGTCGCTCAGATTCAAACGATGCACGTAGCGGAATAGGCCCGTGAACTCACTACGGATACTATCGGTGTCAATAACGATGGCCACTTGGATGCTCGTGGTCGTCGAATTGTGAACCTAGCGAACGCCGTGGATGACCGCGATGCTGTTCCGTTTGGTCAACTAAAGACCATGGACCAGAACTCATGGCAAGCACGGAAGGAAGACTTACAGTTCCGTAATGAGGCTgagaa                                                                                                                      >  1:79521/1‑300 (MQ=255)`  
                                                                                                                                                                                             `aaCCTCCACTGCCCACCGATTGGTTTACTTTTCGGCTGGTTCCATCCCCCGCGCGTATGACCTTAACCTCCCTCAGATTCAAACCATGCACGTAGCGGAAGAGGCCCCTTAACTCACCACGGATACTATCGGTGTCCATAACGATGGTCACCTGGATTCTCGTTGTTGGCCGATTTTTAACCTAGCGAACCCCGTGGATGACCCCGGTTCTTTTCCGTTTTGTCAACTAAAGACCATGGACCAGAACTCATGGCAAGCACGTAATGAAGCCTTACAGTTCCGTAATGAGGCTGAGACTTTc                                                                                                                  <  1:159949/301‑1 (MQ=255)`  
                                                                                                                                                                                             `aaCCTCCACTACCGACCGATTGGTTGACTTTACGGATGGTTCAATCCTCCGCGCGTATGACCTTAACGTCGCTCAGATTCAAACGATGCACGTAGCGGAAGAGGCCCGTGACCTCACTACGGATACTATCGGTGTCAATAACGATGGTCACTTGGATGCTCGTGGTCGTCGAATTGTGAACCTAGCGAACGCCGTGGATGACCGCGATGCTGTTCCGTTTGGTCAACTAAAGACCATGGACCAGAACTCATGGCAAGCACGTAATGAAGCCTTACAGTTCCGTAATGAGGCTGAGACtttt                                                                                                                  >  1:207246/1‑300 (MQ=255)`  
                                                                                                                                                                                               `ccTCCACTACCGACCGATTGGTTGACTTTACGGATGGTTCAATCCTCCGCGCGTATGACCTTAACGTCGCTCAGATTCAAACGATGCACGTAGCGGAAGAGGCCCGTGAACTCACTACGGATACTATCGGTGTCAATAACGATGGTCACTTGGATGCTCGTGGTCGTCGAATTGTGAACCTAGCGAACGCCGTGGATGACCGCGATGCTGTTCCGTTTGGTCAACTAAAGACCATGGACCAGAACTCATGGCAAGCACGTAATGAAGCCTTACAGTTCCGTAATGAGGCTGAGACTTTCAg                                                                                                                >  1:640424/1‑301 (MQ=255)`  
                                                                                                                                                                                                   `cACTACCGACCGATTGGTTGACTTTACGGATGGTTCAATCCTCCGCGCGTATGACCTTAACGTCGCTCAGATTCAAACGATGCACGTAGCGGAAGAGGCCCGTGAACTCACTACGGATACTATCGGTGTCAATAACGATGGTCACTTGGATGCTCGTGGTCGTCGAATTGTGAACCTAGCGAACTCCGTGGATGACCGCGATGCTGTTCCGTTTGGTCAACTAAAGACCATGGACCAGAACTCATGGCAAGCACGTAATGAAGCCTTACAGTTCCGTAATGAGGCTGAGACTTTCAGAAAc                                                                                                            <  1:184823/301‑1 (MQ=255)`  
                                                                                                                                                                                                    `aCTACCGACCGAGTGGTTGAATTTAAGGAAGGTTCAATCCTTCGCGCGTATGACCTTAACGTCGCTCAGATTCAAACGATGCACGTAGCGGAAGAGGCCCGTGACCTCACTACGGATACTATCGGTGTCAATAACGATGGTCACTTGGATGCTCGTGGTCGTCGAATTGTGAACCTAGCGAACGCCGTGGATGACCGCGATGCTGTTCCGTTTGGTCAACTAAAGACCATGGACCAGAACTCATGGCAAGCACGTAATGAAGCCTTACAGTTCCGTAATGAGGCTGAGACTTTCAGAAAcc                                                                                                           <  1:60881/301‑1 (MQ=255)`  
                                                                                                                                                                                                     `cTACCGACCGATTGGTTGACTTTACGGATGGTTCAATCCTCCGCGCGTATGACCTTAACGTCGCTCAGATTCAAACGATGCACGTAGCGGAAGAGGCCCGTGAACTCACTACGGATACTATCGGTGTCAATAACGATGGTCACTTGGATGCTCGTGGTCGTCGAATTGTGAACCTAGCGAACGCCGTGGATGACCGCGATTCTGTTCCGTTTGGTCAACTAAAGCCCATGGACCAGAACTCATGGCAAGCACGTAATGAAGCCTTACAGTTCCGTAATGAGGATGAGACTTTCAGAAACCa                                                                                                          >  1:634010/1‑301 (MQ=255)`  
                                                                                                                                                                                                     `cTACCGACCGATTGGTTGACTTTACGGATGGTTCAATCCTCCGCGCGTATGACCTTAACGTCGCTCAGATTCAAACGATGCACGTAGCGGAAGAGGCCCGTGAACTCACTACGGATACTATCGGTGTCAATAACGATAGTCACTTGGATGCTCGTGGTCGTCGAATTGTGAACCTAGCGAACGCCGTGGATGACCGCGATGCTGTTCCGTTTGGTCAACTAAAGACCATGGACCAGAACTCATGGCAAGCACGTAATGAAGCCTTACAGTTCCGTAATGAGGCTGAGACTTTCAGAAACCa                                                                                                          >  1:284105/1‑301 (MQ=255)`  
                                                                                                                                                                                                     `cTACCGACCGACTGGTTGACTTTACGGATGGTTCAATCCTCCGCGCGTATGACCTTAACGTCGCTCAGATTCAAACGATGCACGTAGCGGAAGAGGCCCGTGAACTCACTACGGATACTATCGGTGTCAATAACGATGGTCACTTGGATGCTCGTGGTCGTCGAATTGTGAACCTAGCGAACGCCGTGGATGACCGCGATGCTGTTCCGTTTGGTCAACTAAAGACCATGGACCAGAACTCATGGCAAGCACGTAATGAAGCCTTACAGTTCCGTAATGAGGCTGAGACTTTCAGAAACCa                                                                                                          >  1:239033/1‑301 (MQ=255)`  
                                                                                                                                                                                                          `cacctttttgtttccTTTACGGTTGTTTCAATCCTCCGCGCGTATGACCTTAACGTCGCTCAGATTCAAACGATGCACGTAGCGGAAGAGGCCCGTGAACTCACTACGGATACTATCGGTGTCAATAACGATGGTCACTTGGATGCTCGTGGTCGTCGAATTGTGAACCTAGCGAACGCCGTGGATGACCGCGATGCTGTTCCGTTTGGTCAACTAAAGACCATGGACCAGAACTCATGGCAAGCACGTAATGAAGCCTTACAGTTCCGTAATGAGGCTGAGACTTTCAGAAACCAAGCgg                                                                                                     <  1:39552/287‑1 (MQ=255)`  
                                                                                                                                                                                                                         `tttACGGATGGTTCAATCCTCCGCGCGTATGACCTTAACGGCGCTCAGATTCAAACGATGCACGTAGCGGAAGAGGCCCGTGACCTCACTACGGATACTATCGGTGTCAATAACGATGGTCACTTGGATGCTCGTGGTCGTCGAATTGTTAACCTAGCGAACGCCGTGGATGACCGCGATGCTGTTCCGTTTGGTCAACTAAAGACCATGGACCAGAACTCATGGCAAGCACGTAATGAAGCCTTACAGTTCCGTAATGAGGCTGAGACTTTCAGAAACCAAGCGGAGGGCTTTAAGAACg                                                                                      <  1:444941/301‑1 (MQ=255)`  
                                                                                                                                                                                                                                 `tGGTTCAATCCTCCGCGCGTATGACCTTAACGTCGCTCAGATTCAAACGATGCACGTAGCGGAAGAGGCCCGTGACCTCACTACGGATACTATCGGTGTCAATAACGATGGTCACTTGGATGCTCGTGGTCGTCGAATTGTGAACCTAGCGAACGCCGTGGATGACCGCGATGCTGTTCCGTTTGGTCAACTAAAGACCATGGACCAGAACTCATGGCATGCACGTAATGAAGCCTTACAGTTCCGTAATGAGGCTGAGACTTTCCGAAACCAAGCAGAGGGCTTTAAGAACgctgtaact                                                                              >  1:382002/1‑293 (MQ=255)`  
                                                                                                                                                                                                                                   `gTTCAATCCTCCGCGCGTATGACCTTAACGTCGCTCAGATTCAAACGATGCACGTAGCGGAAGAGGCCCGTGAACTCACTACGGATACTATCGGTGTCAATAACGATGGTCACTTGGATGCTCGTGGTCGTCGAATTGTGAACCTAGCGAACGCCGTGGATGACCGCGATGCTGTTCCGTTTGGTCAACTAAAGACCATGGACCAGAACTCATGGCAAGCACGTAATGAAGCCTTACAGTTCCGTAATGAGGCTGAGACTTTCAGAAACCAAGCGGAGGGCTTTACGAACGAGTCCAGTAc                                                                            >  1:106059/1‑301 (MQ=255)`  
                                                                                                                                                                                                                                   `gTTCAATCCTCCGCGCGTATGACCTTAACGTCGCTCAGATTCAAACGATGCACGTAGCGGAAGAGGCCCGTGAACTCACTACGGATACTATCGGTGTCAATAACGATGGTCACTTGGATGCTCGTGGTCGTCGAATTGTGAACCTAGCGAACGCCGAGGAGGACCGAGATGCTGTTCCGTTTGGTCAACTAAAGACCATGGACCAGAACTCATGGCAAGCACGTAATGAAGCCTTACAGTTCCGTAATGAGGCTGAGACTTTCAGAAACCAAGCGGAGGGCTTTAAGAACGAGTCCAggaa                                                                            >  1:202731/1‑298 (MQ=255)`  
                                                                                                                                                                                                                                   `gTTCAATCCTCCGCGCGTATGACCGTAACGTCGCTCAGATTCAAACGATGCACGTAGCGGAAGAGGCCCGTGACCTCACTACGGATACTATCGGTGTCAATAACGATGGTCACTTGGATGCTCGTGGTCGTCGAATTGTGAACCTAGCGAACGCCGTGGATGACCGCGATGCTGTTCCGTTTGGTCAACTAAAGACCATGGACCAGAACTCATGGCAAGCACGTAATGAAGCCTTACAGTTCCGTAATGAGGCTGAGACTTTCAGAAACCAAGCGGAGGGCTTTAAGAACGAGTCCAGTAc                                                                            <  1:535237/301‑1 (MQ=255)`  
                                                                                                                                                                                                                                    `ttCAATCCTCCGCGCGTATGACCTTAACGTCGCTCAGATTCAAACGATGCACGTAGCGGAAGAGGCCCGTGACCTCACTACGGATACTATCGGTGTCAATAACGATGGTCACTTGGATGCTCGTGGTCGTCGAATTGTGAACCTAGCGAACGCCGTGGATGACCGCGATGCTGTTCCGTTTGGTCAACTAAAGACCATGGACCAGAACTCATGGCAAGCACGTAATGAAGCCTTACAGTTCCGTAATGAGGCTGAGACTTTCAGAAACCAAGCGGAGGGCTTTAAGAACGAGTCCAGTAcc                                                                           >  1:196368/1‑301 (MQ=255)`  
                                                                                                                                                                                                                                    `tgcAATCCTCCGCGCGTATGACCTTAACGTCGCTCAGATTCAAACGATGCACGTAGGGGAAGAGGCCCGTGAACTCACTACGGATACTATCGGTGTCAATAACGATGGTCACTTGGATGCTCGTGGTCGTCGAATTGTGAACCTAGCGAACGCCGTGGATGACCGCGATGCTGTTCCGTTTGGTCAACTAAAGACCATGGACAAGAACTCATGGCAAGCACGTAATGAAGCCTTACAGTTCCGTAATGAGGCTGAGACTTTCAGAAACCAAGCGGAGGGCTTTAAGAACGAGTCCAGTatc                                                                           <  1:100298/299‑3 (MQ=255)`  
                                                                                                                                                                                                                                        `aaagagacagcgTATGACCTTAACGTCGCTCAGATTCAAACGATGCACGTAGCGGAAGAGGCCCGTGACCTCACTACGGATACTATCGGTGTCAATAACGATGGTCACTTGGATGCTCGTGGTCGTCGAATTGTGAACCTAGCGAACGCCGTGGATGACCGCGATGCTGTTCCGTTTGGTCAACTAAAGACCATGGACCAGAACTCATGGCAAGCACGTAATGAAGCCTTACAGTTCCGTAATGAGGCTGAGACTTTCAGAAACCAAGCGGAGGGCTTTAAGAACGAGTCCAGTACCAACg                                                                       <  1:289978/292‑1 (MQ=255)`  
                                                                                                                                                                                                                                         `tgtgaataagagacagccTTAACGTCGATCAGATTCAAACGATGCACGGAGCGGAATAGGTGCGTGAACTCACTACGGATACTAGAGGTGTCAATAACGATGGTCACTTGGATGCTCGTGGTCGTCGAATTGTGAACCGAGCGAACGCCGTGGATGACCGCGATGCGGTTCCGTTTGGTCAAATAAAGACCATGGACCAGAACTCATGGCAAGCACGTAATGAAGCCTTACAGTTCCGTAATGAGGCTGAGACTTTCAGAAACCAAGCTGAGGGCTTTAAGAACGAGTCCAGTACCAACGc                                                                      <  1:123197/285‑1 (MQ=255)`  
                                                                                                                                                                                                                                         `ccctccGCGCGTATGACCTTAACGTCGCTCAGATTCAAACGATGCACGTAGCGGAAGAGGCCCGTGAACTCACTACGGATACTATCGGTGTCAATAACGATGGTCACTTGGATGCTCGTGGTCGTCGAATTGTGAACCTAGCGAACGCCGTGGATGACCGCGATGCTGTTCCGTTTGGTCAACTAAAGACCATGGACCAGAACTCATGGCAAGCACGTAATGAAGCCTTACAGTTCCGTAATGAGGCTGAGACTTTCAGAAACCAAGCGGAGGGCTTTAAGAACGAGTCCAGTACCAACGc                                                                      <  1:587001/300‑1 (MQ=255)`  
                                                                                                                                                                                                                                                    `tATGACCTTAACGTCGCTCAGATTCAAACGATGCACGTAGCGGAAGAGGCCCGTGACCTCACTACGGATACTATCGGTGTCAATAACGATGGTCATTTGGATGCTCGTGGTCGTCGAATTGTGCACCTAGCGCACGACGTGGATGACCGCGATGCTGTTCCGTTTGGTCAACTAAAGACCATGGACCAGAACTCATGGCAAGCACGTAATGAAGCCTTACAGTTCCGTAATTAGGATGAGACTTTCAGAAACCAAGCGGAGGGCTTTAAGAACGAGTTCAGTAACACCGCTACGAACACaa                                                           >  1:370478/1‑301 (MQ=255)`  
                                                                                                                                                                                                                                                       `gACCTTAACGGCGCTCAGATTCAAACGATGCACGTAGCGGAAGAGGCCCGTGAACTCACTACGGATACTATCGGTGTCAATAACGATGGTCACTTGGATGCTCGTGGTCGTCGAATTGTGAACCTAGCGAACGCCGTGGATGACCGCGATGCTGTTCCGTTTGGTCAACTAAAGACCATGGACCAGAACTCATGGCAAGCACGTAATGAAGCCTTACAGTTCCGTAATGAGGCTGAGACTTTCAGAAACCAAGCGGAGGGCTTTAAGAACGAGTCCAGTACCAACGCTACGAACACAAAGc                                                        <  1:331600/301‑1 (MQ=255)`  
                                                                                                                                                                                                                                                        `aCCTTAATGTCGCTCAGATTCAAACGATGCACGTAGCGGAAGAGGCCCGTGAACTCACTACGGATACTATCGGTGTCAATAACGATGGTCACTTGGATGCTCGTGGTCGTCGAATTGTGAACCTAGCGAACGCCGTGGATGACCGCGATGCTGTTCCGTTTGGTCAACTAAAGACCATGGACCAGAACTCATGGCAAGCACGTAATGAAGCCTTACAGTTCCGTAATGAGGATGAGACTTTCAGAAACCAAGCGGAGGGCTTTAAGAACGATTCCAGGACCAACGCTACGAACACAAAGCa                                                       >  1:336424/1‑301 (MQ=255)`  
                                                                                                                                                                                                                                                        `aCCTTAACGTCGCTCAGATTCAAACGATGAACGTAGCGGAAGAGGCCCGTGAACTCAATACGGATACTATCGGTGTCAAGAACGATGGTCACTTGGATGCTCGTGGTCGTCGAATTGTGAACCTAGCGAACGCCGTGGATGACCGCGATGCTGTTCCACTTAGTAAACTAAAGACCATGGCCCAGAACTCATGGCAAGCACGTAATGAAGCCTTACAGTTCCGTAATGAGGCTGAGACTTTCAGAAACCAAGCGGAGGGCTTTAAGAACGAGTCCAGTACCAACGCTACGAACACAAAGCa                                                       <  1:617217/301‑1 (MQ=255)`  
                                                                                                                                                                                                                                                            `tAACGTCGCTCAGATACAAACGATGCACGTAGCGGAAGAGGCCCGTGACCGCACTACGGATACTATCGGTGTCAATACCGATGGTCACTTGGATGCTCGTGGTCGTCGAATTGTGAACCTAGCGAACGCCGTGGATGACCGCGATGCTGTTCCGTTTGGTCAACTAAAGACCATGGACCAGAACTCATGGCAAGCACGTAATGAAGCCTTACAGTTCCGTAATGAGGCTGAGACTTTCAGAAACCAAGCGGAGGGCTTTAAGAACGAGTCCAGTACCAACGCTACGAACACAAAGCAGTgg                                                   <  1:66339/301‑1 (MQ=255)`  
                                                                                                                                                                                                                                                                   `ccccctatccaaacgttgtcagTAGCGTAAGCCGCCCGTAACCTCACTACGGATACTATCGGTGTCAATTACGTTGGACACTTGGATGCTCGTGGTCGTCGAATTGTGAACCTAGCCAACGCCGTGGATGACCGCGATGCTGTTCCGTTTGGTCAACTAAAGACCATGGACCATAACTCATGGCAAGCACGTAATGAACCCTTACAGTTCCGTAATGAGGCTGAGACTTTCAGAAACCAAGCGGAGGGCTTTAAGAACGAGTCCAGTACCAACGCTACGAACACAAAGCAGTGGCGCGATg                                            <  1:596690/280‑1 (MQ=255)`  
                                                                                                                                                                                                                                                                       `tgATTCAAACGATGCACGTAGCTGAAGAGGCCCGTGAACTCACTACGGATACTATCGGTGTCAATAACGATGGTCACTTGGATGCTCGTGGTCGTCGAATTGTGAACCTAGCGAACGCCGTGGATGACCGCGATGCTGTTCCGTTTGGTCAACTAAAGATCATGGACCAGAACTCATGGCAAGCACGTAATGAAGCCTTACAGTTCCGTAATGAGGCTGAGACTTTCAGAAACCAAGCGGAGGGCTTTAAGAACGAGTCCAGTACCAACGCTACGAACACAAAGCAGTGGCGCGATGAGAc                                        <  1:74209/300‑1 (MQ=255)`  
                                                                                                                                                                                                                                                                        `gATTCAAACGATGCACGTAGCGGAAGAGGCCCGTGAACTCACTACGGATACTATCGGTGTCAATAACGATGGTCACTTGGATGCTCGTGGTCGTCGAATTGTGAACCTAGCGAACGCCGTTGATGACCGCGATGCTGTTCCGTTTGGTCAACTAAAGACCATGGACCAGAACTCATGGCAAGCACGTAATGAAGCCTTACAGTTCCGTAATGAGGCTGAGACTTTCAGAAACAACGCGGAGGGCTTTAAGAACGAGTCCAGTACCAACGCTACGAACACAACTCAGTTGCGCGCTGAGAcc                                       >  1:513803/1‑301 (MQ=255)`  
                                                                                                                                                                                                                                                                             `gacagATGAACGTAGCGGAAGAGGCCCGTTAACTCACTACGGATACTATCGGTGTCAATAACGATGGTCACTTGGATGCTCGTGGTCGTCGAATTGTGAACCTAGCGAACGCCGTAGATGACCGCGATGCTGTTCCGTTTGGTCAACTAAAGACCATGGACAAGAACTCATGGCAAGCACGTAATGAAGCCTTACAGTTCCGTAATGAGGCTGAGACTTTCAGAAACCAAGCGGAGGGCTTTAAGAACGAGTCCAGTACCAACGCTACGAACACAAAGCAGTGGCGCGATGAGACCAAggg                                  <  1:359489/297‑1 (MQ=255)`  
                                                                                                                                                                                                                                                                                   `ggCACGTAGCGGAAGAGGCCCGTGAACGCACTACGGATACTATCGGTGTCAATAACGGTGGTCACTTGGATGCTCGTGGTCGTCGAATTGTGAACCTAGCGAAGGCCGTGGATGACCGCGATGCTGTTCCGTTTGGTCAACTAAAGACCATGGACCAGAACTCATGGCAAGCACGTAATGAAGCCTTACAGTTCCGTAATGAGGCTGAGACTTTCAGAAACCAAGCGGAGGGCTTTAAGAACGAGTCCAGTACCAACGCTACGAACACAAAGCAGTGTCGCGATGAGACAAAGGGTTTCCg                            <  1:272611/300‑1 (MQ=255)`  
                                                                                                                                                                                                                                                                                      `aCGGAGCGGAAGAGGCCCGTGAACTCACTACGGATACTATCGGTGTCAATAACGATGGTCACTTGGATGCTCGTGGTCGTCGAATTGTGAACCTAGCGAACGCCGTGGATGACCGCGATGCTGTTCCGTTTGGTCAACTAAAGACCATGGACCAGAACTCATGGCAAGCACGTAATGAAGCCTTACAGTTCCGTAATGAGGCTGAGACTTTCAGAAACCAAGCGGAGGGCTTTAAGAACGAGTCCAGTACCAACGCTACGAACACAAAGCAGTGGCGCGATGAGACCAAGGGTTTCCgaga                         <  1:627670/301‑1 (MQ=255)`  
                                                                                                                                                                                                                                                                                        `ggaGCGGAAGAGGCCCGTGAACTCACTACGGATACAAGAGGTGTCAATACAGATGGTCACTTGGATGCTCGGGGTCGACGAATGGTGAACCTAGCGAACGCCGTGGATGACCGCGATGCGGTTCCGTTTGGTCAACTAAAGACCATGGACCAGAACTCATGGCAAGCACGTAATGAAGCCTTACAGTTCCGTAATGAGGCTGAGACTTTCAGAAACCAAGCGGAGGGCTTTAAGAACGAGTCCAGTACCAACGCTACGAACACAAAGCAGTGGCGCGATGAGACCAAGGGTTTCAGAGACg                       <  1:295994/299‑1 (MQ=255)`  

                                                                                                                                                                                                                                                                                                            `|`                                                                                                                                                                                                                                                                                                               
`CTCCAATCGTGATTTTAATATCCCGTTTGAGTATCTAGCCCGTAAGTTCGTAGTGGTAACTCTTATTGGTGTAGACCGAAAGGTCCTTACGATTAATACAGACTATCGCTTTGCTACACGTACTACTATCTCTCTGACAAAGGCTTGGGGTCCAGCCGATGGCTACACGACCATCGAGTTACGTCGAGTAACCTCCACTACCGACCGATTGGTTGACTTTACGGATGGTTCAATCCTCCGCGCGTATGACCTTAACGTCGCTCAGATTCAAACGATGCACGTAGCGGAAGAGGCCCGTGACCTCACTACGGATACTATCGGTGTCAATAACGATGGTCACTTGGATGCTCGTGGTCGTCGAATTGTGAACCTAGCGAACGCCGTGGATGACCGCGATGCTGTTCCGTTTGGTCAACTAAAGACCATGAACCAGAACTCATGGCAAGCACGTAATGAAGCCTTACAGTTCCGTAATGAGGCTGAGACTTTCAGAAACCAAGCGGAGGGCTTTAAGAACGAGTCCAGTACCAACGCTACGAACACAAAGCAGTGGCGCGATGAGACCAAGGGTTTCCGAGACGAAGCCAAGCGGTTCAAGAATA  >  NC_001604.1/34668‑35269`

  

Alignment Legend

Aligned base mismatch/match (shaded by quality score): `ATCG/ATCG` `< 3 ≤` `ATCG/ATCG` `< 9 ≤` `ATCG/ATCG` `< 19 ≤` `ATCG/ATCG` `< 34 ≤` `ATCG/ATCG` `< 38 ≤` `ATCG/ATCG`

Unaligned base: `atcg`    Masked matching base: `atcg`    Alignment gap: `‑`    Deleted base: `‑`
